# Chronic Voluntary Ethanol Drinking in Cynomolgus Macaques Elicits Gene Expression Changes in Prefrontal Cortical Area 46

**DOI:** 10.1111/acer.14259

**Published:** 2020-01-25

**Authors:** Nicole A. R. Walter, Christina L. Zheng, Robert P. Searles, Shannon K. McWeeney, Kathleen A. Grant, Robert Hitzemann

**Affiliations:** ^1^ Division of Neuroscience Oregon National Primate Research Center Oregon Health & Science University Beaverton Oregon; ^2^ Department of Behavioral Neuroscience Oregon Health & Science University Portland Oregon; ^3^ Division of Bioinformatics and Computational Biology Department of Medical Informatics and Clinical Epidemiology Oregon Health & Science University Portland Oregon; ^4^ Knight Cancer Institute Oregon Health & Science University Portland Oregon; ^5^ Integrated Genomics Laboratory Oregon Health & Science University Portland Oregon

**Keywords:** EtOH, Brain Gene Expression, Cynomolgus Macaque, Cortex

## Abstract

**Background:**

Genome‐wide profiling to examine brain transcriptional features associated with excessive ethanol (EtOH) consumption has been applied to a variety of species including rodents, nonhuman primates (NHPs), and humans. However, these data were obtained from cross‐sectional samples which are particularly vulnerable to individual variation when obtained from small outbred populations typical of human and NHP studies. In the current study, a novel within‐subject design was used to examine the effects of voluntary EtOH consumption on prefrontal cortex (PFC) gene expression in a NHP model.

**Methods:**

Two cohorts of cynomolgus macaques (*n* = 23) underwent a schedule‐induced polydipsia procedure to establish EtOH self‐administration followed by 6 months of daily open access to EtOH (4% w/v) and water. Individual daily EtOH intakes ranged from an average of 0.7 to 3.7 g/kg/d. Dorsal lateral PFC area 46 (A46) brain biopsies were collected in EtOH‐naïve and control monkeys; contralateral A46 biopsies were collected from the same monkeys following the 6 months of fluid consumption. Gene expression changes were assessed using RNA‐Seq paired analysis, which allowed for correction of individual baseline differences in gene expression.

**Results:**

A total of 675 genes were significantly down‐regulated following EtOH consumption; these were functionally enriched for immune response, cell adhesion, plasma membrane, and extracellular matrix. A total of 567 genes that were up‐regulated following EtOH consumption were enriched in microRNA target sites and included target sites associated with Toll‐like receptor pathways. The differentially expressed genes were also significantly enriched in transcription factor binding sites.

**Conclusions:**

The data presented here are the first to use a longitudinal biopsy strategy to examine how chronic EtOH consumption affects gene expression in the primate PFC. Prominent effects were seen in both cell adhesion and neuroimmune pathways; the latter contained both pro‐ and antiinflammatory genes. The data also indicate that changes in miRNAs and transcription factors may be important epigenetic regulators of EtOH consumption.

## Graphical abstract

Walter et al. used a unique within subject design to detect changes in the brain prefrontal cortex transcriptome associated with chronic ethanol exposure in non‐human primates. The data obtained showed that broad categories of genes were affected, especially those associated with neuro‐immune function, cell adhesion and the extracellular matrix. The data suggest that chronic ethanol exposure has marked functional and structural effects on the prefrontal cortex.

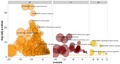

An estimated 16 million people in the United States suffer from an alcohol use disorder (AUD; Substance Abuse and Mental Health Administration (SAaMHS) 2015). Identifying the genetic effects of AUD is important to advance prevention and treatment options in the human population. Beginning with Lewohl and colleagues (2000), there are more than 100 studies using genome‐wide profiling to examine the brain transcriptional features associated with excessive ethanol (EtOH) consumption (see, e.g., Contet, 2012; Farris et al., 2018; Mulligan et al., 2006). These studies have focused on factors associated with risk, individual variation, dependence, and/or chronic consumption. The transcriptional features associated with each of these factors can generally be well isolated in rodent studies; for example, selective breeding can be used to detect risk features in the absence of EtOH exposure (see, e.g., Colville et al., 2017). There are, however, substantial caveats associated with extending the rodent data to the human condition; while the homology between rodent and human for coding genes is greater than 90%, the homology for noncoding RNAs and especially long noncoding RNAs is substantially less (Roux et al., 2017). Further, there are substantial differences between the rodent and human brain including marked differences in proportional cortical volume and the lack of granular cortex in the rodent brain (Saleem et al., 2007 and references therein). A macaque model of excessive alcohol self‐administration (Baker et al., 2014; Grant et al., 2008) can provide increased homology to humans in transcriptional, neuroanatomical, and behavioral features. In contrast to postmortem human studies (Farris and Mayfield, 2014; Lewohl et al., 2000; Warden and Mayfield, 2017), the nonhuman primate (NHP) model (e.g., Iancu et al., 2018a) allows 1 to examine features associated with the initiation and relatively early phase of excessive EtOH consumption, a time window when therapeutic intervention is likely to have the greatest benefit.

Our NHP model of EtOH consumption (Baker et al., 2014; Grant et al., 2008) produces long‐term voluntary consumption over months and individual differences in drinking that reflect a wide range of intoxication and daily drinking patterns similar to humans. Compared to humans, macaques have similar alcohol absorption and metabolism rates (Green et al., 1999; Jimenez et al., 2017). To our knowledge, only 1 NHP brain genome‐wide transcriptome analysis has been completed (Iancu et al., 2018a). This analysis looked at the transcriptional effects of chronic EtOH self‐administration in animals that underwent daily drinking over 12 months of the 22 h/d self‐administration protocol. Iancu and colleagues (2018a) observed postmortem gene coexpression and cosplicing patterns correlated to EtOH intake in the central nucleus of the amygdala and in prefrontal cortical area 32. Membrane and synaptic annotations were significantly overrepresented in the network modules associated with EtOH consumption. What was not known was the expression patterns in these brain areas prior to alcohol ingestion. In the current study, we identified longitudinal transcriptional changes in prefrontal cortex (PFC) area 46 (A46), important in decision‐making in macaques (Gerbella et al., 2013 and references therein). This is the first within‐subject study of gene expression changes in cortical neural circuitry and implicates cellular adaptations in neuroimmune, stress, and extracellular matrix processes in the development of excessive alcohol drinking.

## Materials and Methods

### Animals

**Figure 1:**
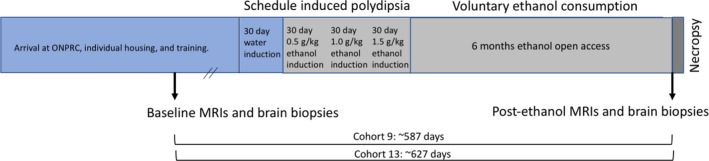
Voluntary EtOH consumption and sample collection timeline.

Two consecutive, replicate cohorts of 11 (cohort 9) and 12 (cohort 13) young adult, male cynomolgus macaques (*Macaca fascicularis*) were housed in quadrant cages (0.8 × 0.8 × 0.9 m) with constant temperature (20 to 22°C), humidity (65%), and an 11‐hour light cycle. Animals had visual, auditory, and olfactory contact with other animals in the protocol. All animals were maintained on positive caloric and fluid balance throughout the experiment, and body weights were recorded weekly. All procedures were conducted in accordance with the Guide for the Care and Use of Laboratory Animals and the NIH guidelines for the care and use of laboratory animal resources and approved by the Oregon National Primate Research Center IACUC. Cynomolgus macaques were ordered from SNBL USA (Everett, WA) and were of Cambodian origin. Monkeys were 5 to 7 years of age (equivalent to 20 to 28 human years) when they first had access to EtOH (See Fig. 1 for overview of procedural timeline). Cohort 9 had 3 controls and 8 EtOH drinkers, and cohort 13 had 3 controls and 9 EtOH drinkers. Detailed drinking data and additional tissue resources are available for these cohorts via the Monkey Alcohol and Tissue Resource Center (Daunais et al., 2014).

### EtOH Subjects

Monkeys were moved into individual housing and then were trained to use operant drinking panels. Once trained, they underwent 3 months of schedule‐induced polydipsia (SIP) to induce EtOH self‐administration in daily 16‐hour sessions, as previously described (Grant et al., 2008). SIP is an effective protocol to establish voluntary EtOH consumption through the use of interval schedules of food delivery. Briefly, banana‐flavored food pellets (1 g) were delivered every 300 seconds (for a fixed time of 300 seconds) until the intended volume of fluid was consumed. After water only SIP, monkeys were induced to increasing volumes of EtOH in 30‐day increments: 0.5, 1.0, and 1.5 g/kg/d (Fig. 1). Following induction, open access (or EtOH self‐administration) began. For each 22‐hour session, water and 4% (w/v) EtOH were concurrently available for 6 months. The subjects received 3 meals (approximately 2 hours apart) of banana pellets each session (day). Each daily session of 22 hours began in the late morning and ended early morning the following day. EtOH was removed for 2 hours each day for cage cleaning, blood draws, and other daily routine procedures. The lighting schedule in the room was 11‐hour light, 13‐hour dark; the lights went off 7 hours after the start of the open‐access session. The details of the open‐access protocol are discussed in Grant and colleagues (2008). The 2 cohorts were run consecutively. EtOH intake in the macaque model is recorded daily with a 0.5‐second resolution.

### Control Subjects

Control subjects were housed in the same room as the EtOH drinking subjects and participated in all experimental manipulations. For the controls, SIP and self‐administration conditions were identical, with the exception that both spouts dispensed water. A maltose dextrin solution (10% in water) was given to the controls to calorically match the drinkers and controls. The dextrin solution was given at the beginning of each daily session by attaching a bottle to the front of the housing cage.

### Biopsy Samples

MRI was used to obtain the stereotaxic coordinates for prefrontal cortical A46 biopsies from the contralateral dominant hemisphere (determined by handedness) from 6 controls and 10 drinkers. Seven drinkers were not biopsied but had sham surgeries. Biopsy samples were 35 to 45 mg wet weight. Samples from cohort 9 were homogenized immediately in Trizol and then frozen at −80°C for future processing; samples from cohort 13 were fresh, frozen on dry ice, and stored at −80°C for future processing. Contralateral A46 biopsy samples after 6 months of drinking were taken from anesthetized animals immediately before necropsy. Before and after samples for cohort 9 were processed simultaneously using a standard Trizol protocol. Before and after samples for cohort 13 were processed simultaneously using a standard Qiagen AllPrep DNA/RNA/miRNA Universal Kit protocol.

### RNA‐Seq

Libraries without strand orientation were prepared using the TruSeq RNA Sample Preparation Kit version 2 (Illumina, San Diego, CA). Libraries were sequenced according to specification on a HiSeq 2500 (Illumina) at the Oregon Health and Science University Massively Parallel Sequencing Shared Resource. PolyA‐selected libraries were multiplexed 4 per lane, yielding an average of 44 million total single‐end reads per sample. FastQC (Babraham Bioinformatics, Babraham Institute, Cambridge, UK) was used for quality checks on the raw sequence data. Reads were aligned to NCBI assembly MF5 (accession GCA_000364345.1) using STAR version 2.5.2b (Dobin et al., 2013) with default parameters except for the following: outFilterMismatchNmax = 2 and outFilterMultimapNmax = 1. Using HTSeq version 0.6.1p1 (Anders et al., 2015) and the MF5 GTF annotation file (33,107 genes), read counts were summarized at the gene level. On average, 85% of reads uniquely mapped to the MF5 genome. Generalized linear models, using DESeq2 (Love et al., 2014), were used to detect differential expression utilizing a blocking design to account for baseline differences within individual animals and individual cohorts (see “Results” for threshold details). RNA‐Seq data are available through NCBI GEO database (accession # pending).

### Gene Ontology Enrichment

GO enrichment was conducted on the EtOH‐affected differentially regulated genes using GOrilla (Eden et al., 2009) with human annotations and TPM ≥ 1 as the background. Significance level was set at FDR *q* < 0.05.

### miRNA Target Enrichment

Enrichment of human miRNA target sites in the up‐ and down‐regulated genes was conducted using miRTarBase (Chou et al., 2018) which is a database of validated microRNA–target interactions. Enrichr (Chen et al., 2013; Kuleshov et al., 2016) was used for the specific enrichment analysis. Subsequently, miRNA Enrichment Analysis and Annotation tool (miEAA; Backes et al., 2016) was used to assess overrepresentation of gene ontology categories in the most connected miRNAs (i.e., those with target sites in the most up‐regulated genes). The 2263 human miRNAs in miRTarBase were used as the reference dataset (i.e., the full human dataset queried for target site enrichment), as well as the full human gene list in miRTarBase.

### Transcription Factor Enrichment

Enrichr (Chen et al., 2013; Kuleshov et al., 2016) and position weighted matrices (PWM) from TRANSFAC (Matys et al., 2006) and JASPAR (Sandelin et al., 2004) were used to assess enrichment of human transcription factor (TF) binding sites in the promoter regions of the identified DEGs. The reference datasets used for enrichment were promoter regions of all human genes in the TRANSFAC and JASPAR databases and the reference TF binding sites therein.

## Results

### Voluntary EtOH Consumption

**Figure 2:**
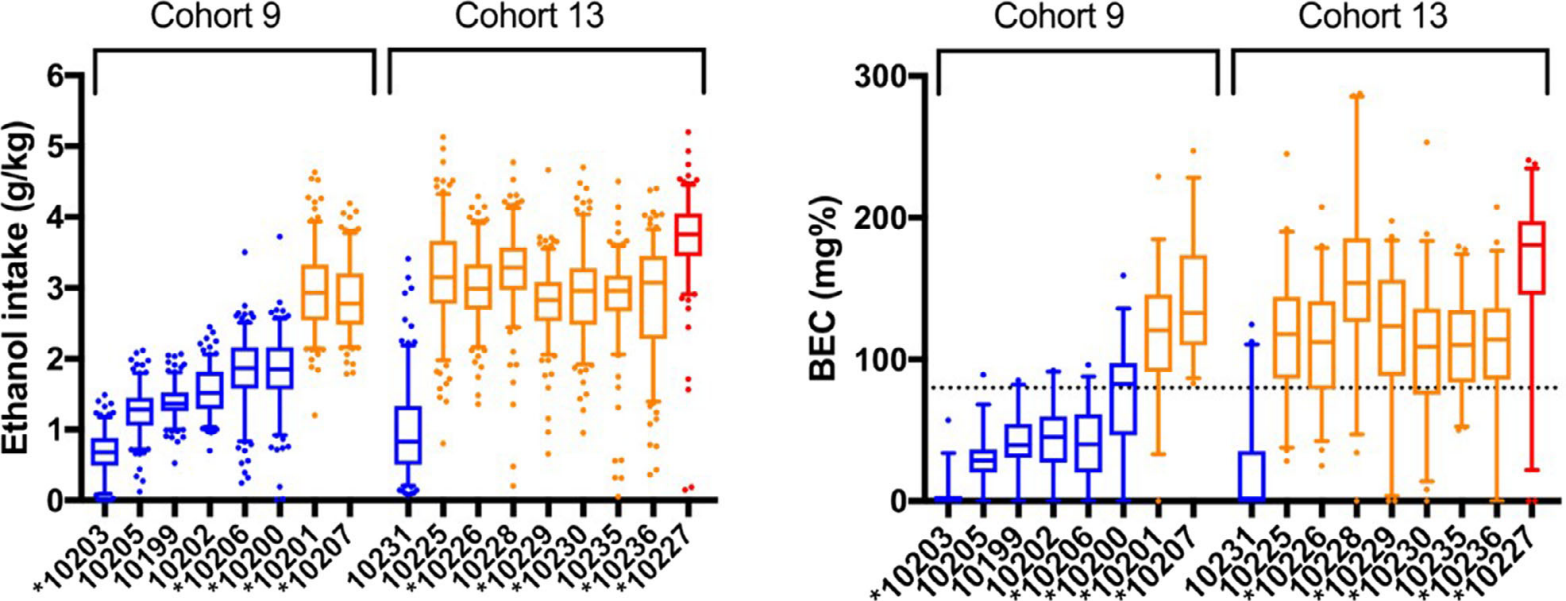
Daily EtOH intake (g/kg) of all the EtOH drinkers in cohorts 9 and 13 over 6 months of EtOH self‐administration. Individual days that fall outside of the 5 to 95% confidence interval are shown. Individuals are colored by drinking category: low (blue), heavy (orange), and very heavy (red). BECs (mg%) were measured every 5 to 7 days at 7 hours into the open‐access session. Dotted line at 80 mg%, which is intoxication level. For both plots, box and whiskers indicate 25 to 75 percentiles and 5 to 95 percentiles, respectively, with line at the median. Subject IDs with an asterisk (*) had brain biopsy samples collected prior to open access.

During 6 months of open access to EtOH, the subjects across both cohorts consumed an average of 0.7 to 3.7 g/kg/d of EtOH (Fig. 2) or an overall average of 2.3 g/kg/d of EtOH. This resulted in average blood EtOH concentrations (BECs) ranging from 6 to 172 mg% (Fig. 2). BECs were measured approximately every 5 days during the 6‐month open‐access period at 7 hours into the daily 22 hours of open access. Due to the timing of the blood sampling, the BECs do not necessarily reflect the total daily intake, but rather the intake pattern over the first 7 hours of daily access. One of 4 drinking categories was assigned to each subject following 6 months of collected data: low, binge, heavy, or very heavy (Fig. 2). These drinking categories are based on data‐driven definitions extracted from previous cohorts that underwent this same consumption protocol (Baker et al., 2014). None of the subjects in these 2 cohorts fit the category for a binge drinker. By chance, most of the low drinkers (LD) were in cohort 9 and most of the high drinkers were in cohort 13. We did account for cohort effect in the analyses. This type of grouping is unpredictable, and there is not a straightforward explanation for this difference. Therefore, the cohorts were analyzed together. The drinking results of the subjects that received a sham surgery rather than a biopsy are included here because it is important to report the drinking results of the full cohort of animals. The gene expression results of these animals are not analyzed here because they did not have a paired before‐EtOH sample; however, the results are included in the GEO submission for further analyses of these cohorts in the future.

### Differential Gene Expression Following 6 Months of Voluntary EtOH Consumption

**Figure 3:**
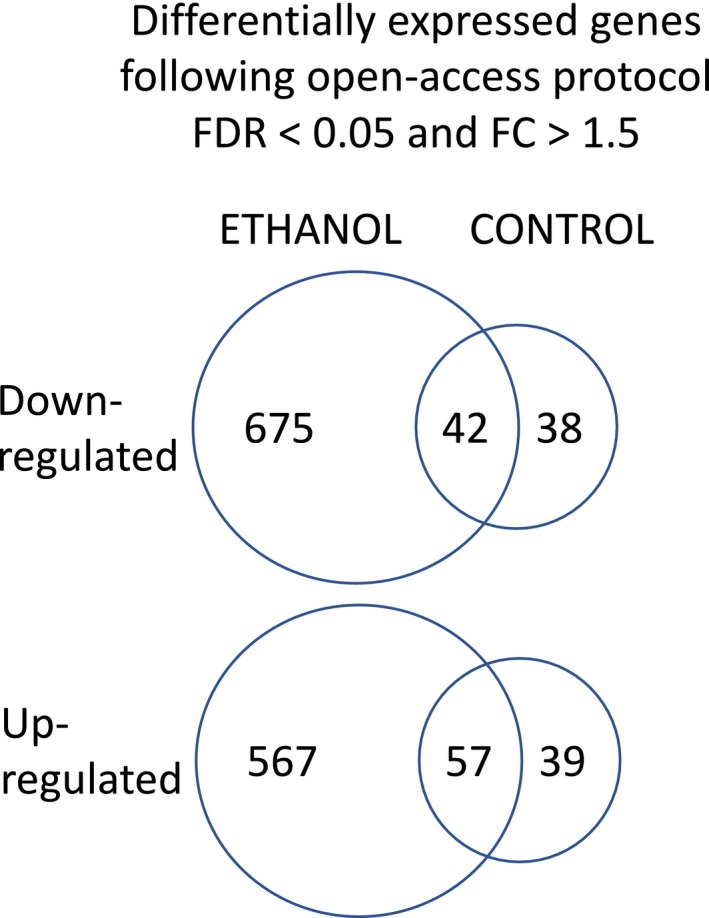
Venn diagram indicating differentially expressed genes (DEGs) following open‐access protocol that reached criteria (*q* < 0.05 and fold change (FC) >1.5) and whether they were identified in EtOH subjects, controls, or both.

A total of 10 drinkers and 6 controls across 2 cohorts were biopsied in A46 prior to EtOH induction (preEtOH biopsy) and again after 6 months (postEtOH biopsy) of voluntary EtOH consumption (or maltose dextrin for the controls; Fig. 1), resulting in a total of 32 RNA‐Seq samples. Utilizing a block design to account for individual and cohort baseline differences, differentially expressed genes identified after alcohol exposure were identified by comparing preEtOH biopsy and postEtOH biopsy samples. In an effort to eliminate confounding effects due to aging, environment, surgeries, etc., differentially expressed genes identified from the comparison of pre‐ and postEtOH biopsies from nonalcohol exposed animals (controls) were subtracted from those identified above. A total of 29,961 coding and noncoding genes are annotated in the cyno MF5 genome. The genes queried were limited to the 15,344 that have average transcripts per million (TPM) ≥1 across all samples. Using FDR *q* < 0.05 and a gene expression fold change (FC) of at least 1.5, we identified 567 up‐regulated and 675 down‐regulated DEGs in the EtOH drinkers before versus after EtOH open access (Fig. 3). These genes were not differentially expressed in the controls. Thirty‐nine up‐regulated and 38 down‐regulated DEGs specific to the controls were identified. A total of 57 up‐regulated and 42 down‐regulated DEGs were differentially expressed in both the controls and the drinkers (Fig. 3). A full list of gene counts (transcripts per million) and differential expression calculations for the controls and the EtOH drinkers are included in Table S1. Only the DEGs specific to EtOH drinkers were further analyzed.

### GO Enrichment of DEGs

The 1,242 DEGs following EtOH consumption were enriched in GO Process [GO:0006955 immune response (*q* = 1.7 × 10^−13^), GO:0006954 inflammatory response (*q* = 1.5 × 10^−11^), GO:0007155 cell adhesion (*q* = 1.0 × 10^−5^)], GO Function [GO:0038023 signaling receptor activity (*q* = 1.9 × 10^−10^), GO:0019955 cytokine binding (*q* = 1.8 × 10^−4^)], and GO Component [GO:0005886 plasma membrane (*q* = 3.7 × 10^−9^), GO:0031012 extracellular matrix (*q* = 2.1 × 10^−2^)]. The gene lists for each ontology category are primarily down‐regulated genes following EtOH consumption. The full list of GO enrichment categories is found in Table S2, which includes the genes in each annotation.

### Transcription Enrichment of DEGs

We examined the transcriptional changes related to the up‐ or down‐regulated genes. Because miRNA target sites for rhesus and human are ~90% conserved (Xu et al., 2013), we used experimentally validated target sites for human microRNAs (miRNAs). The 567 up‐regulated genes were enriched in target sites for 197 human miRNAs (*q* < 0.05). The top 10 miRNAs (enriched based on *q* value) are listed in Table 1a as examples of miRNAs potentially affected by chronic EtOH exposure. The full list of significantly enriched miRNA target sites, including gene lists, is found in Table S3. There was no significant enrichment for miRNA target sites in the down‐regulated genes.

**Table 1:** (a) Top 10 (Based on Adjusted *p* Value) Human miRNAs That Have Significant Number of Target Sites in Our Up‐Regulated Genes. Overlap = [Number of Genes Up‐regulated in Our Dataset With a Binding Site for This miRNA)]/[Total Number of Genes in miRTarBase With Target Sites for This miRNA in the 3′ Untranslated Region]. (b) Top 11 Most Connected miRNAs With the Number of Genes Having 3′UTR Binding Sites for the miRNAs

(a) Most enriched miRNAs			(b) Most connected miRNAs	
miRNA	Overlap	*Q* value	miRNA	# of genes
**hsa‐miR‐192‐5p**	65/993	2.53E−07	**hsa‐miR‐192‐5p**	65
hsa‐miR‐30b‐5p	38/416	2.53E−07	hsa‐miR‐93‐5p^a^	67
**hsa‐miR‐30a‐5p**	53/733	2.97E−07	**hsa‐miR‐30a‐5p**	53
hsa‐miR‐30e‐5p	32/347	2.85E−06	hsa‐miR‐16‐5p	63
**hsa‐miR‐215‐5p**	50/755	4.76E−06	**hsa‐miR‐215‐5p**	50
hsa‐miR‐190a‐3p	45/648	4.76E−06	hsa‐miR‐106b‐5p^a^	60
hsa‐miR‐5011‐5p	45/653	4.76E−06	hsa‐miR‐17‐5p^a^	60
hsa‐miR‐1277‐5p	44/624	4.76E−06	hsa‐miR‐20a‐5p^a^	56
hsa‐miR‐101‐3p	33/393	4.76E−06	hsa‐miR‐155‐5p	53
hsa‐miR‐548i	23/202	4.76E−06	hsa‐miR‐519d‐3p	51
			hsa‐miR‐20b‐5p^a^	51

a Part of the mir‐17 family. The 3 miRNAs in bold are included in both lists.

The most connected miRNAs have validated target sites in 50 or more genes up‐regulated in the EtOH drinkers (Table 1b). A total of 222 (40%) of the 567 up‐regulated genes have a 3′UTR target site for at least one of the 11 most connected miRNAs, and 52 of the genes are associated with 5 or more of the miRNAs (see Table S3). The top 10 overrepresented genes with binding sites for the top connected miRNAs are *DNAJB4(10), KLF10(9), PHF6(9), ZBTB35(9), FAM126B(8), HSPA4L(8), ITGA2(8), SPRED1(8), VPS13C(8), and XIAP(8)*, with the number of top miRNA binding sites in parentheses*.* Overall, for the 197 miRNAs that have significantly represented target sites (*q* < 0.05) in our 567 EtOH up‐regulated genes, each miRNA has validated target sites for a minimum of 5 and a maximum of 67 genes. A total of 385 (68%) of the up‐regulated genes have at least 1 overrepresented miRNA target site.

Overrepresentation analysis of gene ontology categories using miEAA for the 11 most connected miRNAs are listed in Table S3; the annotations associated with 9 or more of the miRNAs are listed (*N* = 465). Annotations of interest are highlighted in Table S3 and include GO0002224 Toll‐like receptor signaling pathway (*q* = 6.0 × 10^−4^), GO0007155 cell adhesion (*q* = 6.5 × 10^−4^), GO0001666 response to hypoxia (*q* = 1.8 × 10^−3^), GO0006965 immune response (*q* = 1 × 10^−3^), GO0005518 collagen binding (*q* = 6.5 × 10^−4^), and GO0005525 GTP binding (*q* = 6 × 10^−3^).

Significant enrichment for transcription factor binding sites in DEGs was also detected with binding motifs for 14 and 6 human transcription factors (TF) significantly (*q* < 0.05) enriched in the down‐ and up‐regulated genes, respectively (Table 2). The list of significantly enriched (*q* < 0.05) TF binding sites, including gene lists, is found in Table S4. Of the 6 TF sites enriched in up‐regulated genes, none were differentially expressed. The most enriched transcription factor (*KLF11*) in the down‐regulated genes was down‐regulated only in the controls (DE *q* = 4.9 × 10^−4^). The TF associated with the down‐regulated genes clustered into 2 groups (Table 2), those significant at 10^−8^ or better (*N* = 4) and those significant at ≥10^−4^ (*N* = 10). Associated with the first group were 149 unique genes, and 50 of these genes were associated with all 4 TF. Annotations for the genes associated with the 149 unique genes are found in Table S4. Annotations for the 149‐gene grouping included cell adhesion (*q* < 1 × 10^−5^), immune response (*q* < 6 × 10^−5^), cell motility (*q* < 2 × 10^−2^), plasma membrane (*q* < 1 × 10^−3^), and extracellular matrix (*q* < 3 × 10^−2^; Table S4).

**Table 2:** Transcription Factors (TFs) That Have Significant Number of Target Sites in Our Up‐ or Down‐Regulated Genes

Term	Overlap	Adjusted *p*‐value
TF binding sites overrepresented in up‐regulated genes		
JUND (human)	80/1380	1.93E−07
MYB (human)	76/1409	5.98E−06
POU1F1 (human)	70/1408	2.83E−04
TBP (human)	104/2486	1.82E−03
HNF1B (human)	10/90	1.30E−02
CBEPB (human)	102/2598	1.67E−02
TF binding sites overrepresented in down‐regulated genes		
Group 1		
KLF11 (human)	103/1388	6.53E−12
KLF4 (human)	104/1485	9.47E−11
ZNF148 (human)	104/1591	4.01E−09
CACYBP (human)	91/1350	1.25E−08
Group 2		
MZF1 (human)	79/1389	1.72E−04
SNAI1 (human)	78/1428	5.68E−04
SNAI2 (human)	78/1428	5.68E−04
TCF3 (human)	78/1428	5.68E−04
NFE2 (human)	127/2681	1.16E−03
IKZF1 (human)	73/1373	2.10E−03
ELF3 (human)	76/1488	4.84E−03
SREBF2 (human)	70/1361	6.65E−03
KLF13 (human)	72/1453	1.45E−02
SP1 (human)	69/1406	2.27E−02

Overlap = [number of genes up or down‐regulated in our dataset (FDR < 0.005 + Fold Change > 1.5)]/[total number of genes in TRANSFAC and JASPAR with motifs for this TF in the promoter region].

The second grouping of 10 TF was associated with 329 unique genes. A total of 135 of these genes are also in the group of 149 noted above. Annotations for the 194 genes found only in the second grouping of TF included immune system process (*q* < 4 × 10^−12^), regulation of immune response (*q* < 9 × 10^−12^), cytokine‐mediated signaling pathway (*q* < 2 × 10^−6^), signaling receptor activity (*q* < 4 × 10^−6^), Toll‐like receptor binding (*q* < 7 × 10^−3^), and extracellular region (*q* < 6 × 10^−5^; Table S4).

## Discussion

Two cohorts of young adult, male cynomolgus macaque monkeys achieved a range of stable, daily voluntary EtOH consumption with 6 months of open access to EtOH. Based on body weight and volume consumed, the drinkers ranged from a low average of 3 (human equivalent) drinks per day to a high average of 15 drinks per day. The overall average across the 2 cohorts was about 9 drinks per day. These animals established drinking patterns similar to previous monkey cohorts, and these patterns mimic those found in the humans with AUD (Baker et al., 2014). Detailed daily data on these cohorts and previous cohorts are available on the Monkey Alcohol and Tissue Resource Center Web site (MATRR.com). Based on previously defined categories (Baker et al., 2014), these 2 cohorts had a distribution of 7 LD, 9 heavy drinkers (HD), and 1 very heavy drinker.

Because we collected longitudinal samples from the same animals before and after voluntary EtOH consumption, we were able to adjust for individual baseline differences in gene expression. This paired longitudinal analysis in controls (*n* = 6) and drinkers (*n* = 10) allowed us to identify baseline differences in gene expression and to identify those gene expression changes that are specifically the result of voluntary EtOH consumption. This approach allowed the identification of 1242 DEGs specifically associated with EtOH consumption, highlighting the neuroplasticity of the brain following 6 months of voluntary EtOH consumption. The up‐regulated genes were a diverse set showing a significant enrichment in 2 membrane‐associated annotations (Table S2). Genes in these categories included cadherins and protocadherins (*CDH10*,* CDH19*,* PCDH18*, and* PCDH20*), receptors and receptor subunits (*CHRM2*,* DRD5*,* GABRA1*,* GABRA4*,* GABRG2*,* GLRB, HTR2A*,* HTR2C*, and* NPY5R*), and a cluster of G‐protein receptors (*GPR19, 22, 52, *and* 63*). Cadherins and protocadherins have been implicated as having key roles in EtOH preference (Colville et al., 2017). Variants in *CHRM2* have been implicated as correlates to the age of onset of AUDs in adolescents and young adults (Chorlian et al., 2013). The evidence that *DRD5* has a role in AUDs is limited (Hack et al., 2011). Enoch and colleagues (2012) found that the expression of *GABRG2* was down‐regulated in both the brains of alcoholics and cocaine addicts. Some but not all studies suggest an association between polymorphisms in *HTR2A* and AUDs (see Cao et al., 2014). Mottagui‐Tabar and colleagues (2004) found some evidence of *HTR2C* promotor variants as alcoholism risk factors; however, to our knowledge this has not been confirmed or refuted in more contemporary studies. The cluster of orphan G‐protein receptors has no known association with EtOH phenotypes. *GPR19* is a potential receptor for adropin which is involved in the regulation of water intake. *GPR22* has linked to the regulation of ciliary function which has been shown to be associated with binge EtOH consumption (Iancu et al., 2018a; Verleyen et al., 2014). *GPR52* has been linked to the regulation of striatal function (Song et al., 2018). A deletion on human chromosome 6 that affects *GPR63*,* NOUFA4*, and *KLHL32* has been associated with Tourette’s syndrome and obsessive–compulsive disorder (Hooper et al., 2012).

The up‐regulated genes were significantly enriched in a number of miRNA binding sites, an association that was absent in the down‐regulated genes. Eleven of the miRNAs were the most connected and had validated target sites in 50 or more up‐regulated genes. Overrepresentation analysis of gene ontology categories using miRNA enrichment analysis revealed a broad representation of GO annotation categories including the extracellular matrix which has been previously identified as a risk category for binge EtOH consumption (Iancu et al., 2018a); however, none of the drinkers in this study met the criteria for binge consumption. Of particular interest was the observation that the overrepresentation analysis revealed an association with immune process‐related categories including Toll‐like receptor signaling pathway and immune response. Four previous studies identified EtOH specific miRNA changes in human alcoholics compared to control subjects (Lewohl et al., 2011; Mamdani et al., 2015; Manzardo et al., 2013; Wang et al., 2013). Specifically, Manzardo and colleagues (2013) looked at miRNA expression from frontal cortex of human alcoholics and identified 13 miRNAs up‐regulated in subjects with AUD and 2 miRNAs down‐regulated. In our 567 genes that were up‐regulated following 6 months of EtOH consumption, we identified enrichment in target sites for 2 miRNAs also identified by Manzardo and colleagues (2013): hsa‐miR‐375 and hsa‐miR‐379. These were both found to be up‐regulated in alcoholic humans. Lewohl and colleagues (2011) identified 35 up‐regulated miRNAs in human alcoholic PFC samples. We identified 8 of those miRNAs (hsa‐miR‐1, hsa‐miR‐144, hsa‐miR‐153, hsa‐miR‐101, hsa‐miR‐374b, hsa‐miR‐140, hsa‐miR‐586, and hsa‐miR‐580) as having target sites in our list of up‐regulated genes. Mamdani and colleagues (2015) performed coexpression analyses on mRNAs and miRNAs in nucleus accumbens of postmortem alcoholic subjects. Of the 25 miRNA hubs they identified, we found enrichment for target sites in our up‐regulated genes for 4 of them (hsa‐miR‐375, hsa‐miR‐132, hsa‐miR‐361, and hsa‐miR‐4760). Several of the most connected miRNAs also have previous evidence of being affected by EtOH in rodent studies (e.g., mmu‐miR‐16‐5p, mmu‐miR‐16‐5p, mmu‐miR‐93‐5p, mmu‐miR‐17‐5p, mmu‐miR‐155‐5p, mmu‐miR‐30a‐5p; Darcq et al., 2015; Gorini et al., 2013; Lippai et al., 2013; Nunez et al., 2013; Osterndorff‐Kahanek et al., 2018).

It is interesting to note that for the 3 human studies with overlap, the miRNAs were up‐regulated, which usually leads to down‐regulation of mRNA expression; however, the genes we identified were up‐regulated. Temporal shifts in expression in miRNAs affected by EtOH have been observed in mouse at 0 hour versus 8 hour versus 120 hour postEtOH exposure (Osterndorff‐Kahanek et al., 2018). The temporal variation in the miRNAs could explain the difference in direction of the gene expression changes we observe with regard to previously identified EtOH‐affected human miRNAs. In our protocol, the postEtOH biopsies were collected on what would have been a regular voluntary drinking day.

The genes differentially expressed specifically in the drinking macaques have a rich annotation structure and include immune response, inflammatory response, cell adhesion, signaling receptor activity, cytokine binding, plasma membrane, and extracellular matrix. There is now robust evidence of relationships between neuroimmune signaling and AUDs (e.g., Coleman and Crews, 2018; Cui et al., 2014; Harris and Koob, 2017). Neuro‐inflammatory mechanisms are associated with both the risk for and individual variation in excessive EtOH consumption (e.g., Hitzemann et al., 2017; Iancu et al., 2018a). Although these immune categories were primarily associated with the down‐regulated genes, this does not necessarily imply that chronic EtOH consumption has down‐regulated the neuroimmune system and in fact the available data would suggest otherwise (Erickson et al., 2019 and references therein). Examining the genes associated with the annotation of cytokine signaling reveals both proinflammatory (*IL1R1*,* IL2RG*,* IL17RA*, and* CD74*) and antiinflammatory (*IL6R* and *IL10RA*) receptor genes suggesting that chronic EtOH consumption may have established a new balance between the opposing forces. The actual inflammatory status in A46 at the time of sample acquisition is unknown. Nor it is known what cell type(s) are associated with a particular cytokine signal. However, there is ample evidence that chronic EtOH consumption has a marked effect on the expression of cytokine ligands and receptors (Erickson et al., 2019; Montesinos et al., 2016; Warden et al., 2019).

As noted above, the differentially expressed genes were enriched in genes (*N* = 47) associated with the extracellular matrix (ECM). Genes with this annotation included 4 collagens (*COL1A1*,* COL6A2*,* COL18A1*, and* COL23A1*) and 2 matrix metaloproteases (*MMP9 *and* MMP25*). Collagens and the ECM were previously identified as associated with the selection for the High‐Drinking‐In‐the Dark mouse lines (Iancu et al., 2018b). Alcohol and other drugs of abuse can have marked effects on ECM constituents (reviewed in Lubbers et al., 2014). EtOH is known to affect the brain expression of tPA (or *PLAT*), *MMP*9, *BCAN* & *NCAN,* and *TSP2* & *TSP4* (Bahi and Dreyer, 2012; Coleman et al., 2011; Pawlak et al., 2005; Risher et al., 2015; Wright et al., 2003). Previously, we emphasized (Iancu et al., 2018a) the potential interactions between the ECM and neuroimmune processes, and we repeat that emphasis here. For example, Seo and colleagues (2008) have shown that collagens can induce an inflammatory response in microglia.

Although the down‐regulated genes were not enriched in miRNA binding sites, there was a significant enrichment in TF binding sites. The TF associated with the down‐regulated genes clustered into 2 groups: those significant at 10^−8^ or better (*N* = 4) and those significant at ≥10^−4^ (*N* = 10). None of the TFs associated with these binding sites met our criteria for significant down‐regulation; however, for many they were trending in the right direction (See Table S1). Not surprisingly, the annotations for the down‐regulated genes associated with these TFs (75% of the total) showed significant enrichment in genes associated with immune processes and the ECM.

Some of the TF identified in the current study have been previously found to be associated with alcohol‐related phenotypes. For example, protein levels of Kruppel‐like factor 11 (*KLF11*) have been shown to increase in the postmortem PFC of subjects with alcohol dependence (Udemgba et al., 2014). Further binge drinking increased *KLF11* expression in the rat frontal cortex (Duncan et al., 2016). EtOH has been shown to decrease myeloid zinc finger 1 (*MZF1*) transcription, and *MZF1* is a transcription factor involved in EtOH induced decreases in bone remodeling and fracture healing (Driver et al., 2015). *MZF1* was significantly down‐regulated following 6 months of EtOH consumption, and the set of down‐regulated genes were significantly enriched in *MZF1* binding sites with 83 down‐regulated genes containing a *MZF1* binding site(s) in their promoter regions.

In conclusion, the data presented here are, to our knowledge, the first example of using a within‐subject design to assess the effects of chronic EtOH consumption on brain gene expression. The statistical advantages of this approach are obvious and especially welcome when working with NHPs. The small sample sizes precluded dividing the drinkers according to their average daily consumption; however, we retain the expectation that the transcriptional signature for the low and HD will differ and is likely to differ across some of the dimensions described here such as neuroimmune response. A key observation noted here, as well as previously (Iancu et al., 2018a), is that both pro and antiinflammatory immune‐related genes are affected. In addition, we have again noted a strong effect on ECM‐related elements. Finally, we consider that an interaction between immune and ECM gene networks is likely critical to understanding the transcriptional effects of chronic EtOH exposure.

## Funding

This work was supported by National Institute of Health grants P60AA010760 (RH, KG, SM, CZ, RS, NW), R24AA019431 (KG), and AA013484 (RH).

## Conflict of interest

The authors declare that they have no competing financial interests.

## Author contributions

NW, KG, and RH conceived and designed the study. RS contributed to RNA‐Seq via MPSSR Core at OHSU. SM, CZ, and NW contributed to gene expression analyses. NW, RH, and CZ prepared the manuscript.

## Supporting information


**Table S1.** Summary of Gene Expression Data.

**Table S2.** Gene Ontology (GO) of Alcohol Affected Genes.

**Table S3.** miRNAs Associated with Alcohol Affected Genes.

**Table S4.** Transcription Factors Associated with Alcohol affected Genes.
